# Seroconversion for cytomegalovirus infection in a cohort of pregnant women in Québec, 2010–2013

**DOI:** 10.1017/S0950268815003167

**Published:** 2015-12-21

**Authors:** V. LAMARRE, N. L. GILBERT, C. ROUSSEAU, T. W. GYORKOS, W. D. FRASER

**Affiliations:** 1 Infectious Diseases Division, Department of Pediatrics, Centre hospitalier universitaire Sainte-Justine, Université de Montréal, Montréal, Québec, Canada; 2 Public Health Agency of Canada, Ottawa, Ontario, Canada; 3 Department of Social and Preventive Medicine, Université de Montréal, Montréal, Québec, Canada; 4 Department of Microbiology and Immunology, Université de Montréal, Montréal, Québec, Canada; 5 Division of Clinical Epidemiology, Research Institute of the McGill University Health Centre, Montréal, Québec, Canada; 6 Department of Epidemiology, Biostatistics and Occupational Health, McGill University, Montréal, Québec, Canada; 7 Department of Obstetrics and Gynecology, Centre de recherche de CHUS, Université de Sherbrooke, Sherbrooke, Québec, Canada

**Keywords:** Cytomegalovirus, infectious disease epidemiology, pregnancy, prevention, serology

## Abstract

Cytomegalovirus (CMV) is the leading cause of congenital infection and non-genetic sensorineural hearing loss in children. There are no recent data on the incidence of CMV infection during pregnancy in Canada. This present study was undertaken to determine the seroprevalence of CMV IgG antibodies and the rate of seroconversion in a cohort of pregnant women in the province of Québec, Canada. We used serum samples and questionnaire data collected as part of the 3D Pregnancy and Birth Cohort Study (2010–2013) conducted in Québec, Canada. CMV IgG antibodies were determined in serum samples collected at the first and third trimesters. Associations between independent variables and seroprevalence were assessed using logistic regression, and associations with seroconversions, by Poisson regression. Of 1938 pregnant women tested, 40·4% were seropositive for CMV at baseline. Previous CMV infection was associated with: working as a daycare educator, lower education, lower income, having had children, first language other than French or English, and being born outside Canada or the United States. Of the 1122 initially seronegative women, 24 (2·1%) seroconverted between their first and third trimesters. The seroconversion rate was 1·4 [95% confidence interval (CI) 0·9–2·1]/10 000 person-days at risk or 3·9 (95% CI 2·5–5·9)/100 pregnancies (assuming a 280-day gestation). The high proportion of pregnant women susceptible to CMV infection (nearly 60%) and the subsequent rate of seroconversion are of concern.

## INTRODUCTION

Cytomegalovirus (CMV) is a common congenital infection. Reported birth prevalence rates range from 0·18% to 2·0% in Western Europe and from 0·44% to 6·2% in the United States [1]. The only published Canadian study, conducted in Hamilton, Ontario, between 1973 and 1976, reported a birth prevalence of 0·42% [2].

Congenital CMV infection (cCMV) leads to long-term sequelae including sensorineural hearing loss and cognitive and motor deficits. Based on data pooled from 15 studies from Western Europe, USA, Canada and Japan, it was estimated that 13% of children with cCMV infection will present specific symptoms at birth and, of these, 40–48% will have permanent sequelae. Of children with asymptomatic cCMV infection, it was estimated that 13·5% will develop long-term sequelae [3].

Cumulative evidence to date suggests that primary CMV maternal infection during pregnancy will result in a transmission of the virus to the fetus in 40% of cases, whereas 1% of women who are already infected before pregnancy (known CMV IgG+) will transmit the virus, either through reactivation or re-infection [1]. The risk of transmission from mother to child seems to increase throughout the stages of pregnancy [4] but, when the fetus is infected earlier in gestation, there is an increased risk of sequelae [5, 6]. When fetal infection is the result of maternal primary infection, the clinical outcome for the infant (and later, the child) is, in general, poorer than when it is the result of reactivation or re-infection (i.e. when the mother is already CMV IgG+) [1, 7].

CMV is shed in urine and saliva, and transmission occurs through contact with these fluids [8]. It can also be acquired sexually [9]. Risk factors associated with CMV infection include having young children, especially if they are attending daycare [10] and working in contact with young children in a non-hospital setting (e.g. daycare) [8, 11].

There are no population-level data on the seroprevalence of CMV IgG antibodies in the Canadian population. A survey of 206 daycare educators in Toronto, Ontario (97% females) found seroprevalence rates of 56% in those aged <30 years and 65% in those aged ⩾30 years [12]. In 2001, a survey of female daycare educators in Montréal, Québec obtained seroprevalence rates of 45%, 57% and 67% in those aged 20–29, 30–39 and 40–49 years, respectively [13]. The US National Health and Nutrition Examination Survey III (NHANES III, 1988–1994) reported seroprevalence rates of 47·3%, 54·4%, 59·7% and 69·8% in females aged 12–19, 20–29, 30–39 and 40–49 years, respectively [14]. Similarly, there are no recent, representative data on the incidence of CMV infection in pregnant women or on the birth prevalence of cCMV infection. Of 56 initially seronegative daycare providers (i.e. a known high-risk population) from Toronto, Ontario, seven (12·5%) seroconverted over a follow-up period of ~1 year [12].

The present study was undertaken to determine the seroprevalence of CMV IgG antibodies and the rate of seroconversion in a cohort of pregnant women in the province of Québec, Canada.

## MATERIALS AND METHODS

### Study population

The study population originated from the 3D (Découvrir, Développer, Devenir) Cohort Study [15]. This is a prospective study of 2366 pregnant women followed in eight healthcare centres in three cities in southern Québec, enrolled mostly during their first trimester of pregnancy. The cohort was assembled to study the effect of a range of factors on pregnancy and birth outcomes. A questionnaire documenting sociodemographic information, and medical and obstetric history was administered to the women, and serum samples were collected at up to four time points during pregnancy (i.e. during the first, second, and third trimesters, and at delivery). For the samples used in this study, first trimester samples had been collected between May 2010 and August 2012, and third trimester/delivery samples had been collected between October 2010 and February 2013.

### Serological tests

Semi-quantitative determination of CMV IgG antibodies by ELISA was done using Captia CMV IgG kits (Trinity Biotech, USA) analysed on a Triturus automated system (Grifols). All sera were analysed using kits from the same batch. Women with equivocal results at their first trimester were included in the calculation of baseline seroprevalence rate but excluded from the follow-up.

### Statistical analyses

Data were analysed using SAS software v. 5.1 (SAS Institute, USA). Seroprevalence rates were determined as the number of participants positive for CMV IgG in all those with a valid test result (negative, equivocal or positive). Associations between independent variables and CMV IgG seroprevalence were determined by simple and multiple logistic regressions, and odds ratios (ORs) and 95% confidence intervals (CIs) were calculated. The seroconversion rate was calculated as an incidence rate using, as the denominator, the number of person-days at risk (i.e. the sum of the number of days between the first and last serological tests). The 95% CI of the seroconversion rate was calculated by Ulm's exact method based on the relation between Poisson and *χ*^2^ distributions [16]. Associations between independent variables and CMV seroconversion were determined by Poisson regressions, and relative risks and 95% CIs were calculated. Missing values for independent variables were considered as a separate category for logistic and Poisson regression analyses, so all records were included in all analyses.

### Ethics

The 3D Cohort Study had been initially approved by the Research Ethics Board of the Centre de recherche du Centre hospitalier universitaire Sainte-Justine. The consent form in the 3D study allowed for the subsequent use of data and biobanked serum in other studies such as the current study. The current study was approved by the Research Ethics Board of the Centre de recherche du Centre hospitalier universitaire Sainte-Justine and by that of Health Canada.

## RESULTS

A total of 1938 pregnant women from the original 3D cohort had serum samples available from both their first trimester of pregnancy and from their third trimester or delivery, and were therefore included in this study. Their characteristics are described in Table 1. Of these, 40·4% were seropositive for CMV IgG antibodies based on their first trimester test result.

**Table 1.** Characteristics of study participants from the 3D Pregnancy and Birth Cohort Study, Québec, 2010–2013

	*n*	%
Age, years		
17–24	132	6·8
25–29	631	32·6
30–34	758	39·1
35–47	412	21·3
Marital status		
Married or common law	1835	94·7
Single or divorced	101	5·2
Number of babies ever born		
0	1075	55·5
1	633	32·7
⩾2	230	11·9
Number of children still alive		
0	1089	56·2
1	631	32·6
⩾2	218	11·2
Education		
Secondary not completed	42	2·2
Secondary completed	149	7·7
Post-secondary	525	27·1
University	1206	62·2
Household income, Canadian dollars		
0–29 999	193	10·0
30 000–59 999	364	18·8
60 000–99 999	705	36·4
⩾100 000	586	30·2
First language		
English	87	4·5
French	1334	68·8
Both English and French	14	0·7
Other	501	25·9
Place of birth		
Canada	1303	67·2
USA	12	0·6
Caribbean	55	2·8
Oceania	2	0·1
Africa	183	9·4
Europe	174	9·0
Asia	101	5·2
Mexico and Central/South America	105	5·4

Numbers do not add up to 1938 (i.e. number of pregnant women included in this study) because of missing values for some variables.

Age ⩾35 years, being single or divorced, working as a daycare educator or as an orderly, having secondary education or less, lower household income, having had children (excluding the current pregnancy), first language other than English or French, and being born outside Canada or the United States were associated with a higher risk of being seropositive. Of these factors, all but age, marital status and working as an orderly showed independent associations with seropositivity in multiple logistic regressions (Table 2). When the analysis was restricted to mothers born in Canada or the United States, results were similar except that there was no association at all between age and seropositivity in simple logistic regression (Table 3).

**Table 2.** Cytomegalovirus IgG seroprevalence from the 3D Pregnancy and Birth Cohort Study, Québec, 2010–2013

	*N*	*n*	%	Unadjusted OR (95% CI)	Adjusted OR (95% CI)
Age, years					
17–29	763	283	37·09	Reference	Reference
30–34	758	303	39·97	1·13 (0·92–1·39)	1·03 (0·79–1·32)
35–47	412	195	47·33	1·51 (1·20–1·94)	0·94 (0·68–1·29)
Marital status					
Married or common law	1835	728	39·67	Reference	Reference
Single or divorced	101	54	53·47	1·75 (1·169–2·61)	1·61 (0·99–2·62)
Occupation					
Nurse or midwife	166	58	34·94	0·81 (0·58–1·14)	0·85 (0·57–1·28)
Orderly	36	26	72·22	3·94 (1·89–8·22)	1·65 (0·68–4·00)
Daycare	23	17	73·91	4·29 (1·68–10·94)	4·49 (1·57–12·82)
Other or unknown	1713	681	39·75	Reference	Reference
Education					
Primary or secondary	191	109	57·07	2·04 (1·50–2·78)	1·97 (1·34–2·92)
Post-secondary	525	194	36·95	0·90 (0·73–1·11)	0·94 (0·72–1·24)
University	1206	476	39·47	Reference	Reference
No data	16	3	18·75	0·35 (0·10–1·25)	0·34 (0·07–1·55)
Household income, Canadian dollars					
0–59 999	557	326	58·53	3·79 (2·96–4·86)	1·53 (1·10–2·12)
60 000–99 999	705	243	34·47	1·41 (1·11–1·79)	1·11 (0·84–1·46)
⩾100 000	586	159	27·13	Reference	Reference
No data	90	54	60·00		1·44 (0·81–2·55)
Number of babies ever born*					
0	1075	390	36·28	Reference	Reference
⩾1	863	392	45·42	1·46 (1·22–1·76)	1·36 (1·09–1·70)
Number of children still alive*					
0	1089	399	36·64	Reference	
⩾1	849	383	45·11	1·42 (1·18–1·71)	
First language					
French or English	1435	383	26·69	Reference	Reference
Other	501	399	79·64	10·75 (8·39–13·75)	3·84 (2·76–5·34)
Country of birth					
Canada or USA	1315	324	24·64	Reference	Reference
Other	620	458	73·87	8·65 (6·95–10·76)	3·67 (2·70–4·98)

OR, Odds ratio; CI, confidence interval; *N*, Number of women in category; *n*, number of seropositive women.

* Excluding the current pregnancy

**Table 3.** Cytomegalovirus IgG seroprevalence in women born in Canada or USA from the 3D Pregnancy and Birth Cohort Study, Québec, 2010–2013

	*N*	*n*	%	Unadjusted OR (95% CI)	Adjusted OR (95% CI)
Age, years					
17–29	581	144	24·8	Reference	
30–34	507	125	24·7	0·99 (0·75–1·31)	
35–47	225	55	24·4	0·98 (0·69–1·40)	
Marital status					
Married or common law	1245	296	23·8	Reference	Reference
Single or divorced	70	28	40·0	2·14 (1·30–3·51)	1·67 (0·95–2·93)
Occupation					
Nurse or midwife	116	20	17·2	0·63 (0·381–1·04)	0·71 (0·43–1·19)
Orderly	12	4	33·3	1·51 (0·45–5·05)	1·08 (0·30–3·89)
Daycare	14	8	57·1	4·02 (1·38–11·69)	3·96 (1·31–11·97)
Other or unknown	1173	292	24·9	Reference	Reference
Education					
Primary or secondary	137	61	44·5	2·82 (1·93–4·11)	1·91 (1·24–2·95)
Post-secondary	397	91	22·9	1·04 (0·78–1·39)	0·89 (0·65–1·23)
University	771	171	22·2	Reference	Reference
No data	10	1	10·0	0·39 (0·05–3·10)	0·34 (0·04–2·77)
Household income, Canadian dollars					
0–59 999	275	94	34·2	2·00 (1·43–2·78)	1·46 (0·97–2·19)
60 000–99 999	516	116	22·5	1·11 (0·82–1·51)	1·12 (0·81–1·53)
⩾100 000	489	101	20·7	Reference	Reference
No data	35	13	37·1		
Number of babies ever born*					
0	747	153	20·5	Reference	Reference
⩾1	568	171	30·1	1·67 (1·30–2·15)	1·64 (1·26–2·13)
Number of children still alive*					
0	756	157	20·8	Reference	
⩾1	559	167	29·9	1·63 (1·26–2·09)	
First language					
French or English	1263	299	23·7	Reference	Reference
Other	52	25	48·1	2·99 (1·71–5·22)	2·68 (1·50–4·79)

OR, Odds ratio; CI, confidence interval; *N*, Number of women in category; *n*, number of seropositive women.

* Excluding the current pregnancy.

A total of 1122 participants initially seronegative had either their third trimester (*n* = 1106) or delivery serum sample (*n* = 16) tested. Time intervals between the collection of first trimester and third trimester/delivery samples ranged from 106 to 224 days (mean 148 days). Twenty-four of these women seroconverted (i.e. 2·1%). This yielded a seroconversion rate of 1·4 (95% CI 0·9–2·1)/10 000 person-days at risk, or 3·9 (95% CI 2·5–5·9)/100 pregnancies (assuming a standard gestation period of 40 weeks or 280 days), or 5·1% (95% CI 3·2–7·7) on an annual basis. None of the participants' characteristics documented in the study showed a significant association with seroconversion, although a marginal increase in risk (*P* = 0·0748) was observed in women born outside Canada or the USA (Table 4).

**Table 4.** Cytomegalovirus seroconversions in participants from the 3D Pregnancy and Birth Cohort Study, Québec, 2010–2013

	No. of seroconversions	Rate*	Unadjusted RR (95% CI)	*P*
Age, years				
17–29	12	1·74	1·29 (0·54–3·06)	0·5620
30–34	9	1·35	Reference	
35–47	3	0·99	0·73 (0·20–2·70)	0·6398
Marital status				
Married or common law	23	1·44	Reference	
Single or divorced	1	1·52	1·06 (0·14–7·82)	0·9571
Education				
Primary or secondary	1	0·88	0·72 (0·09–5·47)	0·7466
Post-secondary	9	1·90	1·55 (0·66–3·63)	0·3117
University	13	1·23	Reference	
No data	1	5·16	4·21 (0·55–32·17)	0·1661
Household income, Canadian dollars				
0–59 999	5	1·54	0·88 (0·83–2·53)	0·8116
60 000–99 999	7	1·06	0·61 (0·23–1·56)	0·2996
⩾100 000	11	1·75	Reference	
No data	1	1·99	1·14 (0·15–8·84)	0·8993
Number of babies ever born†				
0	12	1·22	Reference	
⩾1	12	1·77	1·46 (0·65–3·24)	0·3581
Number of children still alive†				
0	12	1·21	Reference	
⩾1	12	1·79	1·48 (0·67–3·30)	0·3347
First language				
French or English	20	1·30	Reference	
Other	4	3·13	2·41 (0·82–7·04)	0·1091
Country of birth				
Canada or USA	18	1·24	Reference	
Other	6	2·87	2·32 (0·92–5·84)	0·0748

RR, Relative risk; CI, confidence interval;

* Rate/10 000 person-days at risk.

† Excluding the current pregnancy.

## DISCUSSION

This is the largest study of CMV infection in a pregnant population in Canada. Moreover, with strict definitions of positive and negative such as those used in this study, and exclusion of equivocal results in the seroconversion calculations, our seroconversion rates are solid and reliable.

In this study, a large proportion (58%) of women was seronegative for CMV during the first trimester of pregnancy, placing them at risk for primary CMV infection. Three-quarters of women born in Canada and the United States were susceptible to primary CMV infection compared to one quarter of those born outside of these countries. This significant difference between the two groups of women (OR 8·65 of being positive for CMV IgG if born outside Canada or USA) remained strong after adjustment for confounders (OR 3·65). The higher CMV seroprevalence observed in mothers born outside Canada is consistent with previous Canadian studies [12, 13].

Several risk factors for higher seroprevalence were found to be consistent with those previously published. CMV seropositivity was significantly more frequent in mothers who had more children [12, 13] and in those with lower socioeconomic status, as expressed by income and education indicators [17, 18].

Working with young children, outside of healthcare settings, is recognized as a high-risk occupation for CMV infection. Indeed, in a study carried out in Toronto, Canada, in the 1990s, an annual seroconversion rate of 12·5% was observed in daycare educators [12]. We did observe a higher CMV seropositivity in daycare workers. In Quebec, under the province's preventive withdrawal/reassignment programme for pregnant and breast-feeding women, pregnant workers whose occupation (such as daycare educators) constitutes a hazard to their health or to the health of their fetus may request to be reassigned or, if this not feasible, to be withdrawn from the workplace [19]. In this study, the small number of initially seronegative daycare educators (*n* = 6) precluded any further analysis in this group.

In contrast with previous research, age presented a more complex picture. In univariate analysis, seroprevalence increased with age, which is consistent with findings from NHANES in the United States [14] but this effect was lost in the multivariate analysis. When age was included in a regression model with other variables such as country of origin, it became less important as a determinant of infection, and when the seroprevalence data were analysed separately in Canada/United States-born women, the seroprevalence did not vary by age group, even in univariate analysis. The lack of increase in seroprevalence with age in this cohort may be explained by the fact that most infection is acquired in infancy and childhood, with a subsequent more gradual increase during adulthood. In the study of daycare workers cited above [13], all study subjects were at increased risk relative to the population as a whole and the age range was wider.

The rate of seroconversion observed in this study [3·9/100 pregnancies (assuming a standard gestation period of 40 weeks or 280 days) or 5·1% on an annual basis] is in the upper range of rates previously observed in pregnant women in the United States and higher than most rates reported from Europe [20]. This relatively high rate of seroconversion during pregnancy in a group of adult women with low rates of seropositivity (40%) raises questions about the source of infection in this population and why these women have not seroconverted before. Although we could not answer these important questions with our study, we propose a few hypotheses.

First, there have been major changes in the sociodemographic landscape of the province of Québec over the last 50 years that have likely influenced the epidemiology of CMV. Up until 1950, Québec's birth rate was very high at >30 births/1000. It then plummeted to ~10 births/1000 in 2000 [21, 22]. There was a major influx of women in the workforce, gradually increasing from 1970 onwards [23]. In 1997, the provincial government started an ambitious subsidized daycare programme and that markedly changed the way children were cared for during working hours. Since then, at least 75% of Québec children now attend daycare (either in a daycare facility or in a home-based daycare) before they enter school [24]. There are, therefore, many CMV-seronegative women (60% of our cohort) at risk of exposure to CMV during their second pregnancy. We were unable to assess if there was an increased rate of seroconversion in women who had already had children and who might have attended daycare (and therefore be at higher risk of transmission) because this information was not part of the 3D study questionnaire. In another study in Québec, Canada, among women who had had older children, 82% of these children had been in daycare [25].

Second, there was a trend towards more seroconversion, although not reaching statistical significance, in those seronegative women who were born outside Canada or the United States. This suggests that living in an environment with higher CMV viral burdens (76% of that group was seropositive) may be a risk factor for acquiring CMV. The small number of CMV seroconversions limited our ability to investigate risk factors associated with them.

It is also possible that, in a diverse population of pregnant women, the acquisition of CMV is from so many different types of exposures that no single specific risk can be identified. Some probably acquire the virus through their children, some from a work exposure, and some through their sexual partner. It is disappointing not to be able to clearly identify risk factors. Tailoring a CMV screening programme for high-risk pregnant women requires the identification of key risk factors. In this large cohort, 60% of the women were vulnerable to CMV primary infection. Periodically following the CMV status of all these seronegative women during pregnancy does not seem like a feasible option and we did not identify a group of women suitable for targeted screening based on risk factors for seroconversion. This leaves limited options for a preventive strategy during pregnancy.

Educating women has been proposed as a means of prevention of cCMV [26] but it remains an unproven preventive strategy, as stated recently by the American College of Obstetricians and Gynecologists [27]. In Canada, the current guidelines of the Society of Obstetricians and Gynaecologists of Canada on CMV mention the need for seronegative women to practice good hygiene [28]. Studies by Adler *et al*. demonstrated that, although education of women of childbearing age about CMV was not effective in reducing rates of CMV seroconversion before pregnancy, the education strategy was effective once the women were pregnant [29, 30]. This suggests that motivation to adhere to a hygiene strategy is greater once pregnancy is established.

This study has some limitations. First, the sociodemographic questionnaire was not designed to specifically assess risk factors associated with CMV seroconversion or seropositivity. By the time we developed our study, the enrolment in the 3D study had been completed and sociodemographic data had been collected. However, several participants' characteristics documented in the 3D study were identified as risk factors for CMV infection (e.g. occupation, socioeconomic status, number of children). Still, more details on CMV knowledge, hand-washing and child-rearing practices would have been useful.

Findings from this study may not be generalizable to the entire Québec population because participants were recruited among pregnant women planning to deliver in eight large university hospitals, of which six are in Montréal (the largest city of the province) and the remaining two in two smaller, but still large cities (Québec City and Sherbrooke). These women may be different from those from smaller cities and rural or remote areas with respect to various sociodemographic characteristics found in this study to be associated with CMV infection, such as education, income, and country of origin. Indeed, the percentage of university graduates among study participants (62%) was much higher than that measured in new mothers in recent a population-based survey (35%) [31].

Finally, the main limitation is that we were not able to ascertain if vertical transmission of CMV had actually occurred. The babies born in the 3D study did not have urine or saliva stored that we could have used for CMV testing. We know that 24 women experienced seroconversion. In theory, these are primary infections and we should expect 40% transmission to the fetus: meaning nearly 10 cCMV-infected infants.

In conclusion, our study showed that a significant proportion of women are seronegative for CMV and are at risk of acquiring the infection during pregnancy. Furthermore, nearly 4% of these susceptible women seroconverted in the course of a 280-day pregnancy. This highlights a potential important hidden health issue for children. Further research is needed to ascertain the burden of cCMV infection in the population.
